# Thermoelectric effects in graphene at high bias current and under microwave irradiation

**DOI:** 10.1038/s41598-017-15857-w

**Published:** 2017-11-14

**Authors:** Grigory Skoblin, Jie Sun, August Yurgens

**Affiliations:** 1 Department of Microelectronics and Nanoscience (MC2), Chalmers University of Technology, SE-41296 Gothenburg, Sweden; 2 Key Laboratory of Optoelectronics Technology, College of Microelectronics, Beijing University of Technology, 100124 Beijing, China

## Abstract

We use a split top gate to induce doping of opposite signs in different parts of a graphene field-effect transistor, thereby effectively forming a graphene thermocouple. The thermocouple is sensitive to the electronic temperature in graphene, which can be several hundred kelvin higher than the ambient one at sufficiently high bias current. Combined with the high thermoelectric power of graphene, this allows for i) simple measurements of the electronic temperature and ii) building thermoelectric radiation detectors. A simple prototype graphene thermoelectric detector shows a temperature-independent optical responsivity of around 400 V/W at 94 GHz at temperatures of 4–50 K.

## Introduction

In graphene, the Seebeck coefficient *S* (or thermopower (TEP)) is large, reaching values around 100 µV/K ~ *k*_B_/*e* ^1–3^. This raises the possibility of a new type of radiation detector based on this effect. Indeed, several studies have indicated TEP as a possible mechanism for the way that graphene devices respond to light irradiation^4–9^, for example. However, a number of other explanations are also possible, such as the photovoltaic effect. In addition, placing metal electrodes near graphene provides effective heat reservoirs for non-equilibrium electrons in the graphene, making the TEP effect difficult to detect. Indeed, such heat reservoirs wash out the graphene/metal electronic temperature difference, thereby nullifying the TEP voltage despite the different Seebeck coefficients of the graphene and the metal. Only when the light spot overlaps with both the graphene and its electrode does the corresponding voltage increase significantly^9^. However, in specially designed experiments with dual-gated graphene, the TEP effects in graphene become much clearer^4,7^. Nevertheless, the relatively high transparency of graphene is detrimental to effective light detection.

The electron–phonon coupling in graphene is weak, which can result in significantly different effective temperatures of the electronic and bosonic subsystems when the former is pushed out of equilibrium by a current flowing through the graphene^10^. The electronic temperature *T*_e_ can be assessed by measuring the shot noise, for example. This measurement technique is based on first principles and is straightforward. Several such experiments have shown unambiguously that the electronic temperature can reach several hundred degrees at a high bias current^10^, which was identified as a working mechanism for the intrinsic photo response in graphene^6^. However, the technique requires dedicated setups with low-noise microwave amplifiers and often cryogenic temperatures.

The electronic temperature *T*_e_ is a very steep function of the Joule power dissipated in graphene, especially at small bias^10,11^. Being able to measure small changes in *T*_e_ accurately by shot-noise measurements would be beneficial for building bolometric radiation detectors. The ultimately small volume of graphene would allow for extremely fast bolometric devices, very likely outperforming the state-of-the-art hot-electron bolometers. Unfortunately, however, this technique does not seem to be particular suitable for measuring small changes in the electronic temperature.

Herein, we use the large TEP of graphene to detect a rise in *T*_e_ due to the bias current or external radiation. We make a split top gate to induce doping of different signs (*p*- or *n*-) in two halves of a graphene strip along the direction perpendicular to the current (transverse direction). Any contribution from the longitudinal resistance is largely excluded in this configuration. We also test a simple prototype bolometer by patterning a bow-tie antenna in place of the current-injection contacts. The bolometer shows a clear response to radiation at 94 GHz at both room temperature and 4–50 K, with an estimated optical responsivity of around 400 V/W in the latter case. The noise equivalent power is 20–30 pW/Hz^0.5^ when using an ordinary lock-in measurement system.

## Results

**Figure 1:**
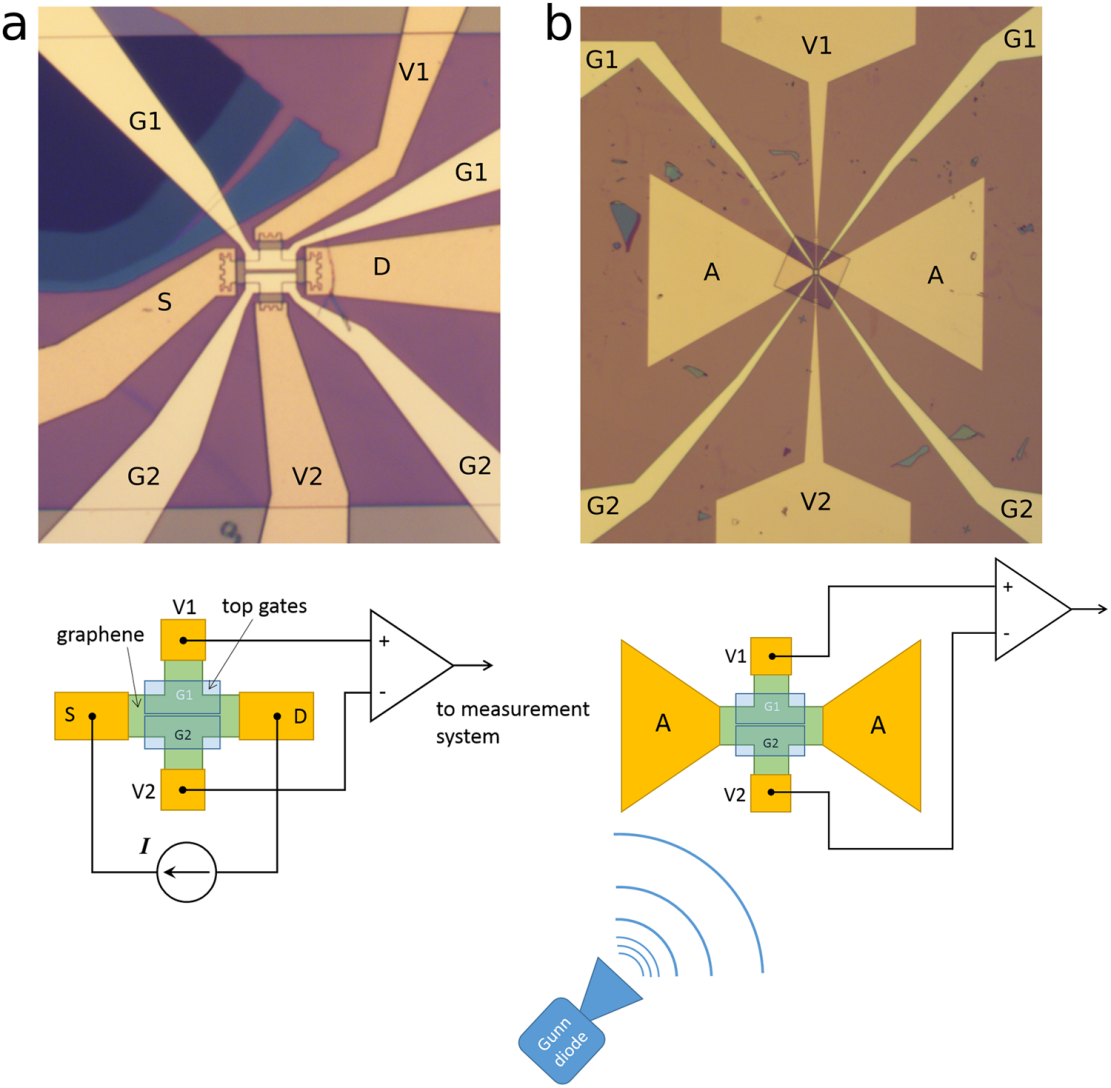
(**a**) An optical image of the sample designed to measure the Joule heating by DC current *I*. There are two leads to each of the top gates G1 and G2 (light yellow). The source (S) and drain (D) electrodes look darker because of an additional layer of Parylene to isolate the graphene edges from contacting the top gates. The edge contacts to the graphene are made as zigzag lines to decrease the overall contact resistances. The graphene bars are 3 µm wide. A thick multilayer graphene flake is seen in the upper-left corner (dark blue). (**b**) As (a) but with a bow-tie antenna (A) instead of the bias (S and D) electrodes. The antenna is 0.86 mm long. (**c**) Schematic of the measurements. Because independent voltages are applied to G1 and G2, graphene doping is different under the corresponding gates, thereby forming a thermocouple made of dissimilar materials, i.e. S1 ≠ S2. The thermocouple can then be used to measure the increased *T*_e_ in the middle. Note that because of the contact geometry, the contribution from the longitudinal (source-to-drain) resistance to the transverse voltage (*V*_2_ − *V*_1_) in zero magnetic field is significantly reduced.

We make our devices using exfoliated graphene encapsulated in Parylene N (see Methods)^12^. The encapsulation offers long-term stability of the device characteristics and is convenient for making low-ohmic edge contacts to graphene. Figure 1 shows typical layouts of the samples studied herein.

### Joule heating

At zero gate voltages and for ideal geometry, we expect no voltage in the transverse direction (i.e. the transverse voltage *V*_**⊥**_ = 0) in zero magnetic field. The electrostatic doping from the back gate and split top gate allows a *p-n* junction to form in the middle of the device where the temperature rises because of Joule heating, which is proportional to the square of the current *I*. Then, because the *p*- and *n* parts of graphene have different Seebeck coefficients, the perpendicular voltage becomes non-zero. Because of its thermal origin, *V*_**⊥**_ must be an even function of *I*; this discriminates a TEP-contribution to *V*_**⊥**_ from those from other sources, such as an inhomogeneous current distribution in the sample.

**Figure 2:**
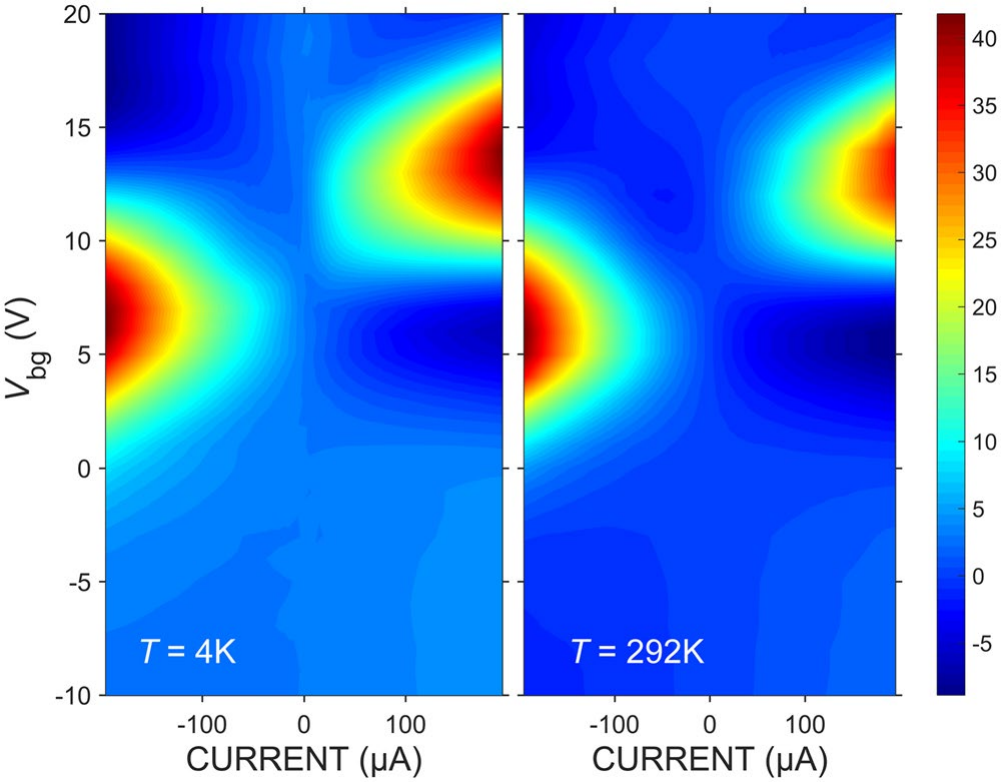
False-colour plots of *V*_**⊥**_ = *V*_2_ − *V*_1_ as a function of current *I* and back-gate voltage *V*_bg_ at constant top-gate voltages *V*_g1_ = −*V*_g2_ = 1 V at *T* = 4 K and 292 K. The colour-bar scale is in millivolts. The self-gating shifts the *V*_**⊥**_ maxima along the ordinate.

**Figure 3:**
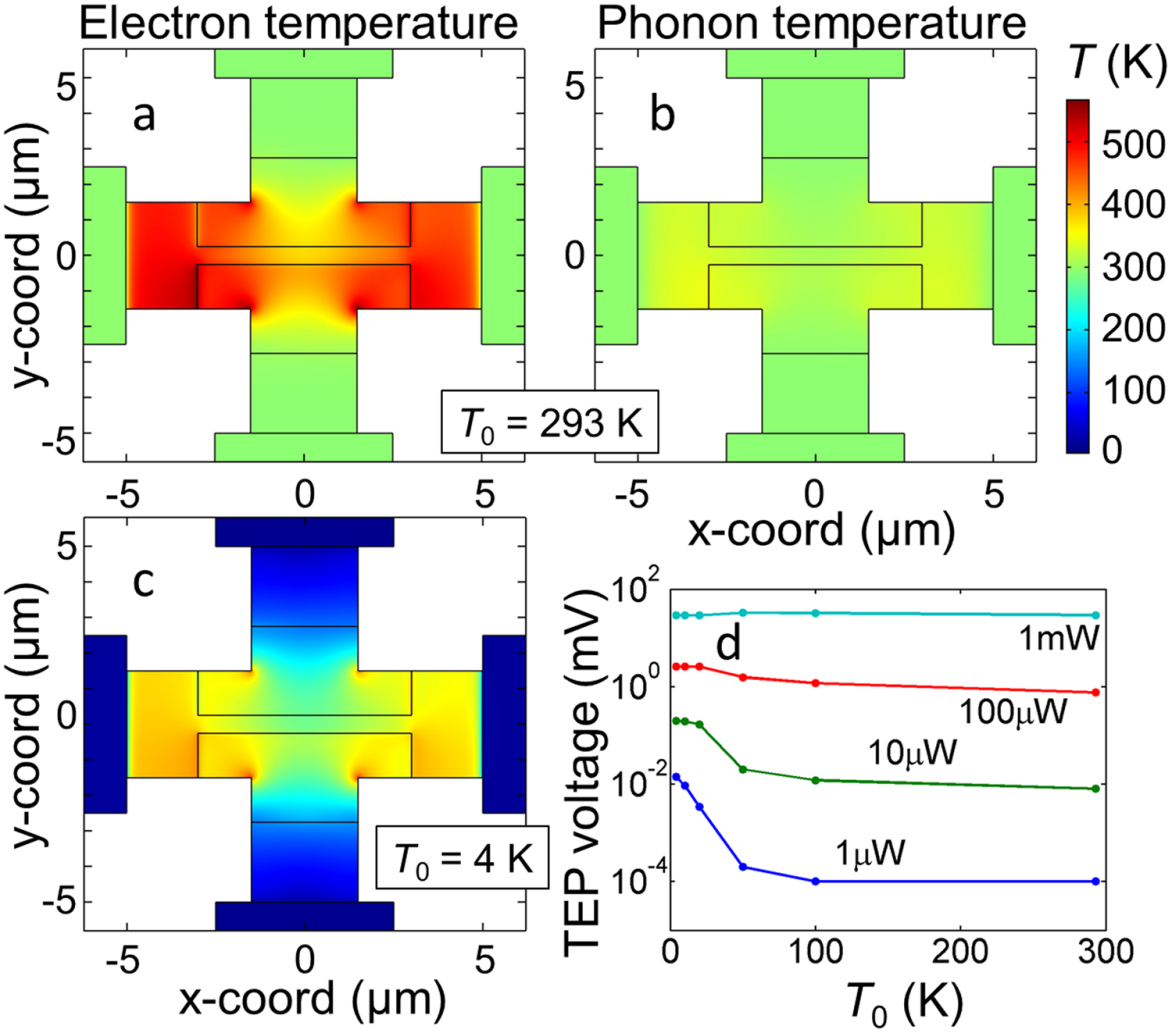
False-colour representations of electron (**a**,**c**) and phonon (**b**) temperature distributions calculated for 1 mW of DC power at ambient temperatures *T*_0_ = 293 K (**a**,**b**) and 4 K (**c**). The thin lines inside the sample denote the split-gate boundaries. The current bias is applied to the horizontal electrodes while the TEP-voltage occurs at the vertical electrodes. (**d**) Temperature dependence of TEP voltage. The total dissipated power is indicated for each curve. The split-gate voltages are ± 3 V for all panels.

We trace *V*_**⊥**_ in the four-dimensional parameter space *V*_**⊥**_ = *V*_**⊥**_(*V*_g1_, *V*_g2_, *V*_bg_, *I*) and present the results in Fig. 2 as a false-colour plot of *V*_**⊥**_(*V*_bg_, *I*) dependence at *T = *4 K and 292 K and at the constant gate voltages *V*_g1_ = −*V*_g2_ = 1 V, which are applied to the two halves of the split top gate; *V*_bg_ is the back-gate voltage. We note that the plots corresponding to *T* = 4 K and 292 K look almost the same, that is *V*_**⊥**_ does not depend on temperature. Moreover, *V*_**⊥**_ is largely positive for both negative and positive *I*. This confirms unambiguously that the TEP effect is responsible for the nonlinear character of the *V*_**⊥**_(*I*) dependence at constant *V*_bg_. The relatively small negative values seen in Fig. 2 can be ascribed to a somewhat inhomogeneous doping in the sample and unevenness of the contact resistivity along the edge-contact lines. The latter is effectively equivalent to a misalignment of the transverse-voltage leads and the appearance of a small contribution from the longitudinal voltage even in zero magnetic field. The maxima of *V*_**⊥**_(*V*_bg_, *I* ≈ ± 0.2 mA) are located at noticeably different (i.e. asymmetric) *V*_bg_. We explain this asymmetry by a self-gating effect when the longitudinal voltage *V*_**||**_ due to a relatively high bias current becomes comparable with the gate voltages. Figure 3 herein and Fig. 7S of the supplementary information show simulation results that include this effect.

### Response to radiation

The increased electronic temperature of graphene when electrical current flows through it and the easiness of TEP readout are both suited to making radiation detectors. What is needed is to attach an antenna structure to convert incoming radiation into electrical current. Figure 1(b) shows an example of such a structure. Comparing it with Fig. 1(a), it is clear that instead of current injection ports (S and D) the graphene now has two metal triangles forming a bow-tie antenna. This antenna receives microwave radiation and focuses the resulting AC current at its middle where the graphene is placed. The two contacts for measuring the voltage that is transverse to the current direction are much thinner than before to minimize their influence on the antenna characteristics. Of course, this increases the overall resistance between the transverse-voltage electrodes and can result in increased Johnson–Nyquist noise. However, the sample layout can be simplified further and the DC TEP readout can be arranged in the longitudinal direction by measuring the DC voltage between the antenna electrodes. The split gate should of course be turned around by 90° to create a *p*-*n* junction crossing the graphene width between the electrodes. The transverse-voltage electrodes can be removed altogether. In this simplified case, the voltage will contain both a DC component from the increased electronic temperature giving rise to a TEP signal and an AC component from the current, which is easy to filter out. Such experiments are under way.

For the second sample (see Fig. 1(b)), we detect the transverse voltage *V*_**⊥**_ resulting from irradiating the sample with microwaves. We use a bow-tie antenna to receive the microwave power and convert it to an AC current in the graphene in the middle part of the structure. Similar to Joule heating by a DC current, the idea is that this AC current will heat up the electronic subsystem in the graphene. We then detect the increased *T*_e_ by using a graphene thermocouple formed by applying voltages of opposite polarity to the split gate. There is no additional current bias in this case.

**Figure 4:**
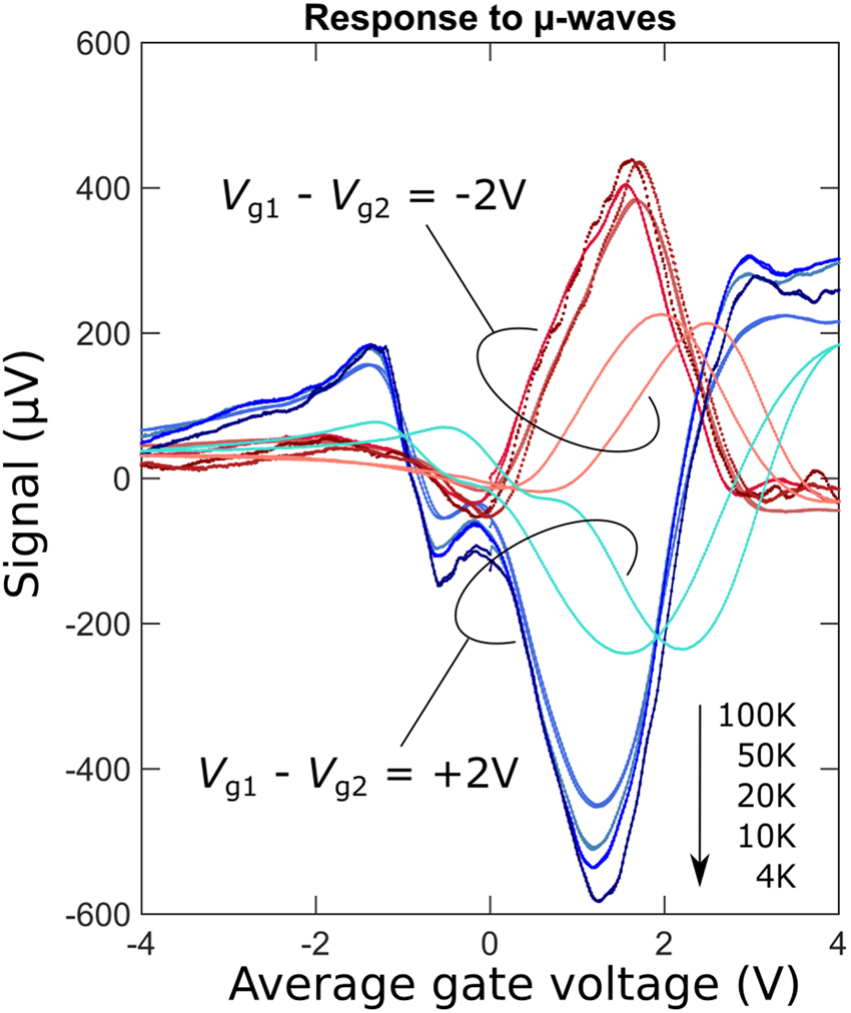
(**a**–**c**) Response to radiation at 94 GHz for sample with antenna (see Fig. 1(b)) as a function of average top-gate voltage (*V*_g1_ + *V*_g2_)/2 at constant voltage difference *δV* = *V*_g1_ − *V*_g2_ = −2 V (shades of red) or +2 V (shades of blue). The arrow indicates the evolution with temperature of the negative-signal maximum at *δV* = +2 V. Note only a weak temperature dependence of the signal despite manifold decrease of the thermoelectric power as the temperature is lowered. The large hysteresis at *T*_0_ = 100 K is due to a charge-carrier freeze-out in the Si substrate.

Figure 4 shows the response to microwave radiation as a function of the average gate voltage *V*_av_ = (*V*_g1_ + *V*_g2_)/2 at different temperatures below 100 K and for the two polarities of *dV*_g_ = *V*_g1_ − *V*_g2_ =  ±2 V. The extrema of the response are near the charge-neutrality point at *V*_av_ = 1.6 V. When *dV*_g_ changes sign, the response signal does the same, indicating that the signal has a thermoelectric origin.

Note that the signal has only a weak temperature dependence over 4–50 K, which is consistent with our DC and low-frequency AC measurements (see Figs 2 and 5S, respectively). The signal is actually somewhat stronger at the lowest temperature despite the linear decrease in thermoelectric power as the temperature is lowered^2^. At about 100 K and above, the signal starts to decrease with temperature down to only roughly 10 µV at room temperature. A clear hysteresis becomes visible when sweeping *V*_av_ in time. Frozen out at low temperature, the charge carriers in the silicon substrate gradually thaw at temperatures above 100 K and start screening the graphene from the microwave radiation. Clearly, one should use more insulating substrates to allow the sensors to operate at room temperature.

Assuming an optical power of roughly 1 µW reaching the sample (see Methods) and a maximum signal value of around 400 µV, we obtain the responsivity *r* = 400 V/W. For a noise floor of roughly 10 nV/Hz^0.5^ for our measurement system, this yields an estimated noise equivalent power of *NEP* ≈ 25 pW/Hz^0.5^ for our detector.

### Simulations

We model our sensors numerically by solving coupled heat-diffusion equations corresponding to phonon and electron systems with temperatures *T*_p_ and *T*_e_, respectively. We use the same sample geometry and materials as in the experiments. We also take into account self gating and two complementary thermoelectric effects (see Methods and supplementary information for assumptions and other details).

We present the calculated temperature distributions in Fig. 3. The electron temperature appears to be significantly higher than the phonon one by over 200 K, in agreement with the shot-noise experiments^10^. The hottest part of the graphene is not in the centre of the sample, suggesting that there is room to improve the sample geometry to maximize the TEP voltage. The Peltier effect leads to noticeable spots inside the graphene that are either colder or hotter than the nearby regions near the vertical edges of the top gate (Fig. 3(a) and (c)). In Fig. 3(d), we present the calculated temperature dependence of the signal at constant power. Despite *S*(*T*) is decreasing when the temperature is lowered^1,2^, the TEP signal stays nearly constant at high power, in agreement with the experimental observations (see Figs 2 and 4). We explain this by the high electron temperature in graphene at low temperature resulting in larger temperature gradients that compensate for the drop in *S*(*T*) (see supplementary information for more details).

## Discussion

Figure 3 shows clearly that the electronic temperature due to Joule dissipation can be very high in graphene, in agreement with earlier experiments^10,11^. Correspondingly, the TEP voltages are large, reaching 100 mV and being easy to measure. At high current, these voltages are nearly the same for all bath temperatures, indicating that the electronic temperature is very high over a wide range of temperature.

A beneficially large TEP in graphene at room temperature allows for a not-so-common readout mechanism in our radiation detectors. Naturally, TEP decreases with decreasing temperature, but the high level of electron heating in graphene at all temperatures more than compensates for this decrease. Indeed, let us make a simple estimation of the minimum detectable power in a 1-Hz band (i.e. the noise equivalent power *NEP*) by assuming the following experimentally verified parameters. The Seebeck coefficient of graphene is *S*(*T*) ~ 100 (*T*/300[K]) µV/K^2^. The radiation power *P* raises the electron temperature *T*_e_ above the bath (phonon) temperature *T*_0_ in accordance with *P* = *β* (*T*_e_^4^ – *T*_0_^4^), where *β* ≈ (0.4–2) mW/(m^2^K^4^) for low temperature and small power^11^. Assuming further that the TEP-voltage measurements are limited by 1/f noise in graphene, namely *N*_SD_ ~ 0.1 µV/Hz^0.5^ ^13^, we obtain the temperature sensitivity of the graphene thermocouple as *δT* ≈ *N*_SD_/*S*(*T*). Then, *NEP*(*T*_0_) ≈ 4*βT*_0_^3^ *δT* ≈ 4*βT*_0_^3^ *N*_SD_/*S*(*T*) ≈ (40–200) (*T*_0_/300)^2^ pW/(µm^2^Hz^0.5^), where we used a linear expansion for small *δT* = *T*_e_−*T*_0_ ≪ *T*_0_ in the limit of fading power. Counter-intuitively but in qualitative agreement with our experiments, this simple estimation shows that the detector should be more sensitive at low temperature despite the decrease of *S*. It also shows that the sensitivity is inversely proportional to graphene area. Our experimental estimation of *NEP* ≈ 25 pW/Hz^0.5^ (see ‘Response to radiation’ above) could be an order of magnitude lower for a reasonably smaller sample. At room temperature, the power law of electron cooling is different, namely *P* = *α* (*T*_e_^3^ – *T*_0_^3^), assuming the so-called super-collision cooling^10^, where *α* ≈ 0.5 W/(K^3^m^2^). A similar estimation of *NEP* for this case gives *NEP*(*T*_0_) ≈ 3*αT*_0_^2^ *δT* ≈ 130 (*T*_0_/300) pW/(µm^2^Hz^0.5^).

A value of *NEP* = 130 pW/Hz^0.5^ for a 1-µm^2^ device is comparable with several other types of room-temperature THz detector, such as Goley cells (~100 pW/Hz^0.5^; see tydexoptics.com) or narrow-band semiconducting bolometers (>10 pW/Hz^0.5^) ^14^. However, the present device is expected to be much faster, with a response rate higher than 200 GHz^15^. If the noise is limited not by 1/f noise but by the lower Johnson noise, we can expect *NEP* values that are another order of magnitude better for graphene detectors with TEP readout. Johnson noise is likely to be more relevant for a thermoelectric open circuit with no bias current.

## Conclusions

We induced inhomogeneous doping in our graphene field-effect transistors to increase the thermoelectric response due to Joule heating by DC or AC currents flowing in the graphene, thereby making the electronic temperature easily accessible. The high thermoelectric power of graphene at room temperature allows for i) simple measurements of the electronic temperature and ii) building radiation detectors with tunable characteristics and low-noise readout. We demonstrated the operation of such a detector by exposing it to microwaves.

## Methods

### Samples

We first exfoliate graphene with the help of sticky tape and lay it down onto 150 nm/90 nm/0.3 mm of Parylene/SiO_2_/Si. A good optical contrast for the graphene dictates the chosen thicknesses. Doped Si at the bottom of this multilayer ‘sandwich’ serves as a back gate. For radiation-detection experiments, we use undoped Si to decrease microwave losses. At room temperature, the electrical conductivity of the undoped Si is still sufficiently high to be able to use it as a gate electrode. The charge-neutrality point in the majority of our devices lies not far from zero doping because Parylene N is non-polar and hydrophobic^12^. Typically, the back-gate voltages needed to reach the charge-neutrality point are less than 10 V for the thickness of dielectric layers given above.

We use e-beam lithography to pattern the graphene structures and their metal contacts after first covering the graphene with the top 90-nm-thick Parylene layer, thereby fully encapsulating the graphene. Oxygen-plasma etching of Parylene and graphene exposes the graphene edges. Lift-off patterning of the Cr/Pd/Au metallization allows these edges to be contacted and the electrodes to be shaped. Finally, we pattern a split top gate making sure it does not contact the graphene edges by covering them with an extra layer of Parylene. The mobility *µ* of the charge carriers estimated from the transfer curves of similar devices but with additional electrodes for measuring the longitudinal resistance is 0.5–1 m^2^/(V s) (see also ref.^12^ for more information about such samples).

### Experiments

For the experiments with microwaves, we glue the sample back-to-back onto a Si hyper-hemispherical lens of 10 mm in diameter and place it into a gas-flow cryostat (Janis PT950) with optical windows made of z-cut quartz. We use a Gunn-diode oscillator at 94 GHz and 30 mW with a conical-horn antenna to generate and direct the microwaves to the sample through the windows. A doped Si wafer in front of the cryostat attenuates the power down to roughly 100 µW. Further attenuation comes from the reflection losses at the window surfaces, from the Si lens and because of geometrical factors. In total, we estimate the optical power reaching the sample to be roughly 1 µW.

A mechanical chopper (SR540) modulates the microwave beam periodically at a frequency of 59 Hz. The periodic response signal from the sample is amplified by an instrumentation amplifier (AMP01, gain 100–1000) and is measured by a lock-in amplifier (SR850) using a 24-dB/Octave filter and a time constant of 0.1–0.3 s (0.26 Hz of the minimum equivalent noise bandwidth). The overall noise floor of the measurement system is roughly 10 nV/Hz^0.5^ at 59 Hz with the sample connected but the cryostat compressor switched off.

### Simulations

We carry out computer simulations of the temperature distribution and calculate the TEP voltage with the help of the COMSOL 5.2 software and assuming the same sample geometry as for the experiments (see Fig. 1). We perform calculations for both high- (~1 mW) and low (~1 µW) dissipated DC power corresponding to our DC-current and microwave-radiation experiments, respectively. We approximate the graphene sheet conductance by *σ* = *μ* [*e* ^2^ *n*_0_^2^ + (*C*_s_ *V*_g_)^2^]^0.5^, where *e*, *n*_0_ (= 3 × 10^11^ cm^−2^), *C*_s_/*e* (= 8.14 × 10^10^ cm^−2^ V^−1^) and *V*_g_ are the electron charge, the residual charge density in graphene, the gate capacitance and the gate voltage, respectively. We assume the conductance and the phonon thermal conductivity *k*_p_ = 600 W/(K m) to be independent of temperature. The phonon energy is dissipated to the Si substrate through the Parylene layer. The weak coupling between the phonon and electron systems depends on the phonon temperature *T*_0_ and the Bloch–Grüneisen temperature *T*_BG_. For *T*_0_ > *T*_BG_, the electron cooling by phonons is cubic in temperature, namely *P*_p-e_ = *α* (*T*_e_^3^ − *T*_0_^3^), whereas for *T*_0_ < *T*_BG_ the cooling power is *P*_p-e_ = *β* (*T*_e_^4^ − *T*_0_^4^), with *α *≈ 0.5 W/(K^3^ m^2^) and *β *≈ 2 × 10^−3^ W/(K^4^ m^2^)^10^. The electron thermal conductivity is taken as *k*_e_ = *γ*(*T*_e_/*T*_0_)^1.6^, where *γ *≈ 6.8 W/(K m)^16^. The Seebeck coefficient for graphene is simulated by the Mott equation, namely *S*(*T*) = *zT*[∂ln(*σ*(*E*))/∂*E*], with the coefficient *z* to adjust the maximum of |*S*| to the experimentally observed 100 µV/K at *T* = 300 K^2^. Although questionable for graphene, the Mott equation reproduces the experimental dependence of *S* on doping and temperature qualitatively well^2^. The Seebeck, Peltier and self-gating effects are included in the calculations (see supplementary information for more details).

The datasets generated during and/or analysed during the current study are are included in this published article (and its Supplementary Information files) or available from the corresponding author on reasonable request.

## Electronic supplementary material

Thermoelectric effects in graphene at high bias current and under microwave irradiation (Supplementary Information)
