# Supplementary material for: Thermoelectric effects in graphene at high bias current and under microwave irradiation
Source: Sci Rep. 2017 Nov 14;7:15542. doi: 10.1038/s41598-017-15857-w (PMC5686182; doi:10.1038/s41598-017-15857-w)
Supplement: Supplementary file 1 — Thermoelectric effects in graphene at high bias current and under microwave irradiation (Supplementary Information) [file 41598_2017_15857_MOESM1_ESM.pdf]

Supplementary information

Thermoelectric effects in graphene at high bias current and under microwave irradiation

G. Skoblin, J. Sun, and A. Yurgens

(Department of Microelectronics and Nanoscience (MC2), Chalmers University of Technology, SE-41296, Gothenburg, Sweden)

1. Self-gating effect

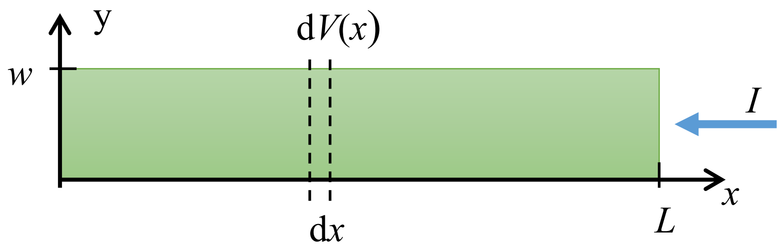

Figure 1S.

Consider a slab of graphene grounded at the left end. The current I, which is injected at the right end, creates the potential distribution $V(x)$ (see Fig. 1S). $V(x)$ would be linear for a constant resistivity of graphene. However, because of the proximity to a gate, this potential induces extra charges, which change the resistivity. The task is therefore to calculate $V(x)$ self-consistently, by solving the following system of equations:

$$\mathrm{d}V(x) = I\rho(x)\frac{\mathrm{d}x}{w}, \quad (1S)$$

$$\rho(x) = \left(\mu\sqrt{C_g^2 V(x)^2 + e^2 n_0^2}\right)^{-1}, \quad (2S)$$

where $\mathrm{d}V(x)$ is the voltage drop across the small section $\mathrm{d}x$ having the sheet resistance $\rho(x)$. μ, C_g, e, and n_0 is the charge mobility, gate capacitance per unit area, electron charge, and residual charge density, respectively.

Combining the above equations and introducing the dimensionless voltage $v(x) = C_g V(x)(en_0)^{-1}$ and current $i = C_g I \mu^{-1}(en_0)^{-2}$, we arrive at a simple differential equation:

$$\mathrm{d}v\sqrt{v^2+1} = i\frac{\mathrm{d}x}{w}, \tag{3S}$$

which has the following nonlinear analytic solution:

$$v_L\sqrt{v_L^2+1} + \ln\left(v_L + \sqrt{v_L^2+1}\right) = 2i\frac{L}{w}\,; \quad v_L = v(L). \tag{4S}$$

In Fig. 2S, we plot the resulting current-voltage (IV) characteristic for the case $L = w$:

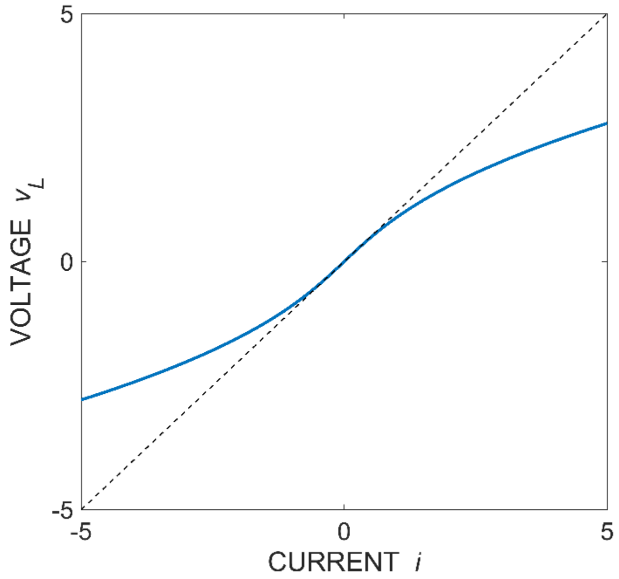

Figure 2S. The dimensionless current-voltage characteristic described by Eq. 4S (solid curve). The dashed line represents the ohmic case $v_L = i$ corresponding to the sheet resistance $\rho = (\mu e n_0)^{-1}$ at the charge-neutrality point.

It is clear from Fig. 2S that deviation from the ohmic law $v_L = i$ is noticeable already at $i \sim 1$ ($I \sim 100\ \mu\mathrm{A}$ for a typical graphene field-effect transistor on SiO_2).

2. AC bias

Here, we test an alternative method for distinguishing the ohmic and thermal contributions to the measured voltage. We use a sample with the layout shown in Fig. 3S. The potential contacts are at the opposite sides of the graphene strip and are shifted along it. If it were measurements in the perpendicular magnetic field, there would be a mixture of the longitudinal- and Hall-effect voltages at these contacts. Instead, there is a mixture of the thermoelectric- and longitudinal voltages in our case of having a *p-n* junction stretching along the graphene strip in its middle. The *p-n* junction is created by sweeping the back-gate voltage V_{bg} while keeping one half of the top split gate grounded. The thin-film leads to another half were unfortunately broken. That part of the gate was therefore left floating and assumed to be at the potential of the back gate.

The TEP voltages are not small at a high current, giving rise to a nonlinearity that can be seen directly in the measured IV characteristics. The nonlinear contribution can then be deduced by

numerically subtracting the linear part of IV. Another way is to bias the sample with a low-frequency current and measure the first- and second harmonics of the signal with the help of a lock-in amplifier.

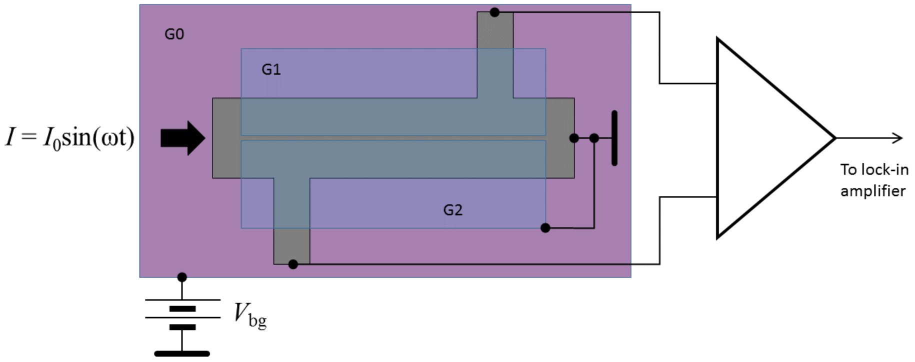

Figure 3S. Layout of the sample used for ac-measurements at 73 Hz. One part of the top split gate (G2) was grounded. Another part was unintentionally left floating and was assumed to be at the same potential V_{bg} as the back gate G0. The first two harmonics of the signal were measured by a lock-in amplifier.

The TEP voltage originates from heating and should be proportional to the square of the current and therefore gives rise to the second harmonic. The longitudinal voltage, including the self-gating effect (see above), will correspond to the first harmonic. Fig. 4S shows results of such measurements conducted at 73 Hz for different current amplitudes as a function of the back-gate voltage. Generally, the curves resemble the gate dependence of the Seebeck coefficient (see e.g. Refs 1 and 2), although with somewhat steeper “shoulders”. The maximum value of the signal matches well the values obtained in the dc measurements.

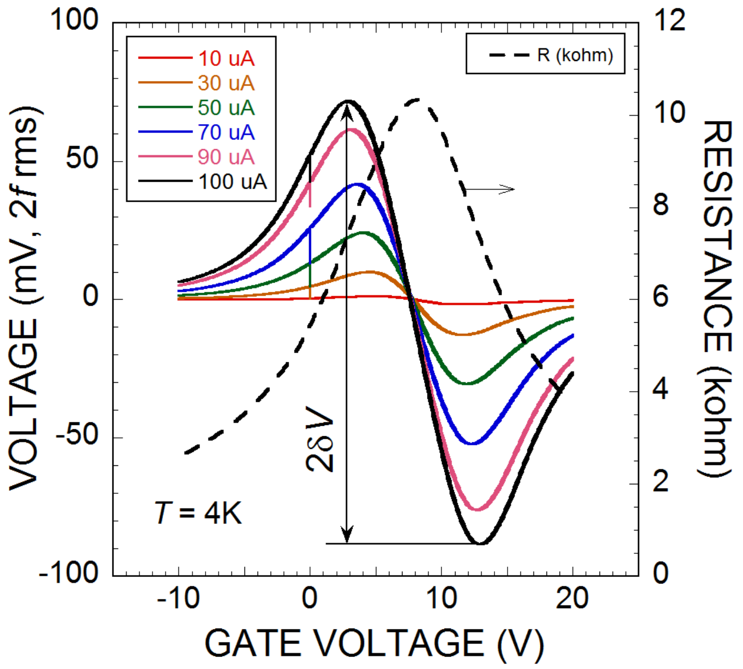

Figure 4S. The second harmonic of the signal at different ac-current levels indicated (solid curves, left ordinate) and the transfer curve obtained from the first harmonic of the signal (dashed curve, right ordinate). Half of the difference between the maximum and minimum of the second harmonic, δV, is taken as a measure of the TEP signal.

Fig. 5S shows that δV (as defined in Fig. 4S) only weakly depends on the bath temperature, in agreement with Fig. 2 of the paper. The Seebeck coefficient S of graphene decreases upon cooling and must be zero at the zero temperature. However, due to a weak electron-phonon coupling, electrons are always hotter than the phonon bath at a finite Joule dissipation. This compensates for the decrease of S. Indeed, the maximum value of S changes linearly with the electronic temperature T (see Ref. 2):

$$S(T) \approx \frac{T}{300} 100 \left[\frac{\mu\mathrm{V}}{\mathrm{K}}\right] = aT. \tag{5S}$$

T depends on the Joule heating P:

$$P = b(T^m - T_0^m), \tag{6S}$$

where m = 3 or 4 has been suggested for different cooling mechanisms at different temperatures (see Refs. 10 and 11). For the expected $T \gg T_0$, the temperature dependence of S should be taken into account, to calculate the measured TEP voltage $u(T)$:

$$u(T) = \int_{T_0}^{T} S(t)dt = \frac{a}{2}(T^2 - T_0^2). \tag{7S}$$

Combining Eqs. 6S and 7S and neglecting T_0 term in Eq. 6S, we arrive at

$$u(T) = \frac{a}{2}\left[\left(\frac{P}{b}\right)^{2/m} - T_0^2\right] \sim \text{const}, \tag{8S}$$

i.e. for $T \gg T_0$, the TEP signal is roughly constant, only slightly decreasing with temperature. This is in qualitative agreement with our experiments, both at the dc- and ac bias (see Figs. 2 and 5S).

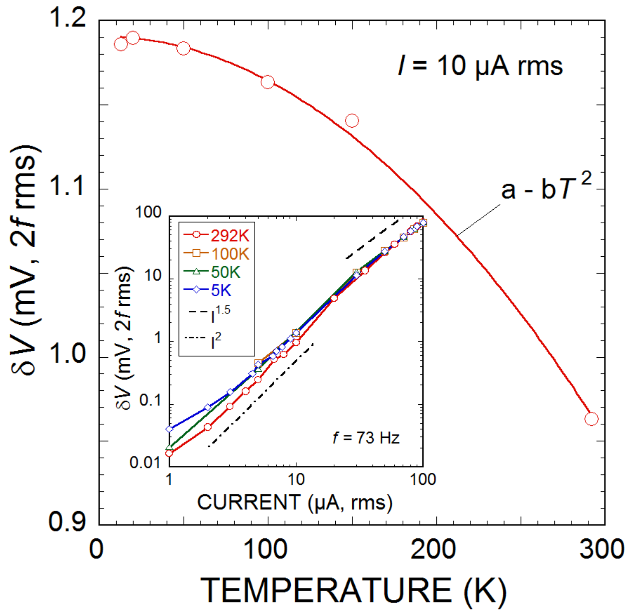

Figure 5S. The temperature dependence of the second harmonic δV defined in Fig. 4S, at the ac current of 10 µA rms and f = 73 Hz. The inset demonstrates the power-law of $\delta V(I)$-dependence at T_0 = 4K.

Further experimental observation is the current dependence $\delta V(I)$ shown in the inset of Fig. 5S. $\delta V(I) \propto I^2$ for small currents and $\delta V(I) \propto I^{1.5}$ for high ones. This dependence can be qualitatively understood from Eq. 8S for $P \propto I^2$ assuming m = 2 or $m \leq 2$ for low- or high I (and T), respectively (see Refs. 10 and 11). Power-law- rather than linear temperature dependence $S(T)$ can be another explanation for this observation (compare e.g. $S(T)$ obtained in Refs. 1 and 2).

3. COMSOL simulations

We use COMSOL 5.3 software to carry out our simulations. The model is two-dimensional with a possibility to take into account the out-of-plane heat flow. The overall goal of these simulations is to find a stationary solution for temperature distributions with the current bias and ambient temperature being the input parameters. The sample geometry used in the program represents a real device with the same dimensions. The mesh is generated automatically with the cell size of 0.1 – 2 µm. We use "Electric currents" and "Heat transfer" components in the simulations with added multiphysics "Thermoelectric effects". The "Heat transfer" component is divided into two parts, one calculating the electron temperature distribution, another – the phonon temperature distribution.

The equations solved in these modules are:

$$\boldsymbol{E} = -\boldsymbol{\nabla} V \tag{9S}$$

$$\boldsymbol{\nabla} \cdot \boldsymbol{J} = 0 \tag{10S}$$

$$\boldsymbol{J} = \sigma(\boldsymbol{E} - S\boldsymbol{\nabla} T_e) \tag{11S}$$

$$\boldsymbol{\nabla} \cdot (k_e \boldsymbol{\nabla} T_e) + \boldsymbol{JE} - T_e \boldsymbol{J}\boldsymbol{\nabla} S - P_{e-ph} = 0 \tag{12S}$$

$$P_{e-ph} + \nabla \cdot (k_{ph} \nabla T_{ph}) - P_{sub} = 0, \quad (13S)$$

where $\boldsymbol{J}$ is the current density, $\boldsymbol{E}$ is the electric field, V is the electric potential, σ is the electric conductivity of graphene, S is the Seebeck coefficient, T_e is the electron temperature, k_e is the electron thermal conductivity, T_{ph} is the phonon temperature, k_{ph} is the phonon thermal conductivity, P_{e-ph} represents the coupling between electrons and phonons, and P_{sub} is the out-of-plane heat flow to the substrate. Eqs. 12S and 13S describe the local heat balance for the two subsystems. Vectors are highlighted in bold.

The conductivity of graphene is calculated according to the formula:

$$\sigma = \mu \sqrt{e^2 n_0^2 + \left(C_{bg}V_{bg} + C_{tg}V_{tg} - (C_{bg} + C_{tg})V\right)^2}, \quad (14S)$$

where μ is the charge-carrier mobility in graphene, e is the elementary charge, n_0 is the residual charge concentration, V_{bg} and V_{tg} are the bottom- and top gate voltages, C_{bg} and C_{tg} are the capacitances per unit area between the graphene and bottom- and top gates, respectively; V is the electric potential in the given point.

The electron thermal conductivity for graphene is $k_e = 6.8 \left(\frac{T_e}{300K}\right)^{1.6}$ W(m K)$^{-1}$ (see Ref. 16). The phonon thermal conductivity is set to 600 W(m K)$^{-1}$. The thermal coupling between the electrons and phonons is discussed in the main paper. The heat transfer from the phonon system of graphene occurs to the golden electrodes and down to the substrate. The characteristics values for gold are taken from the standard sets in COMSOL. There is no much information about thermal conductivity of Parylene in literature. It is therefore assumed to be similar to that of PMMA and have the same temperature dependence (see e.g. Y. Godovsky "Thermophysical Properties of Polymers", Springer Science & Business Media, 1992). The dependence is rather weak in the temperature range 4 – 300 K and can be roughly approximated by the linear function $k_{Par}(T_{ph}) = 1.8 \cdot 10^{-2} + 3.6 \cdot 10^{-4} \cdot T_{ph}$ W(m K)$^{-1}$ adjusted to yield the tabulated value of the thermal conductivity of Parylene at T = 300 K (see e.g. scscoatings.com or www.paryleneengineering.com). The thermal conductivity of Parylene is important only for calculations of phonon temperature.

The boundary conditions are set as following. The initial temperature for the whole system equals the ambient temperature T_0. The edges of graphene are electrically isolated everywhere except the contacts to the electrodes. The far end of one of the horizontal electrodes is grounded; a certain current I_{bias} is pushed through another one. The graphene and the electrodes have thermal contacts with the substrate through 150 nm of Parylene. The electron temperature at the contact interface between the graphene and the electrodes is taken to be equal to the phonon temperature. The device is in vacuum; the substrate that is held at the ambient temperature T_0 is the only way for the heat to outflow from the system.

In Fig. 6S, we show additional simulations of the electron- and phonon temperature in our graphene samples for the ideal case of the very high phonon thermal conductivity $k \approx$ 2500 W(m K)$^{-1}$. Qualitatively, the results are nearly the same as for the smaller k, illustrated by Fig. 3 of the paper.

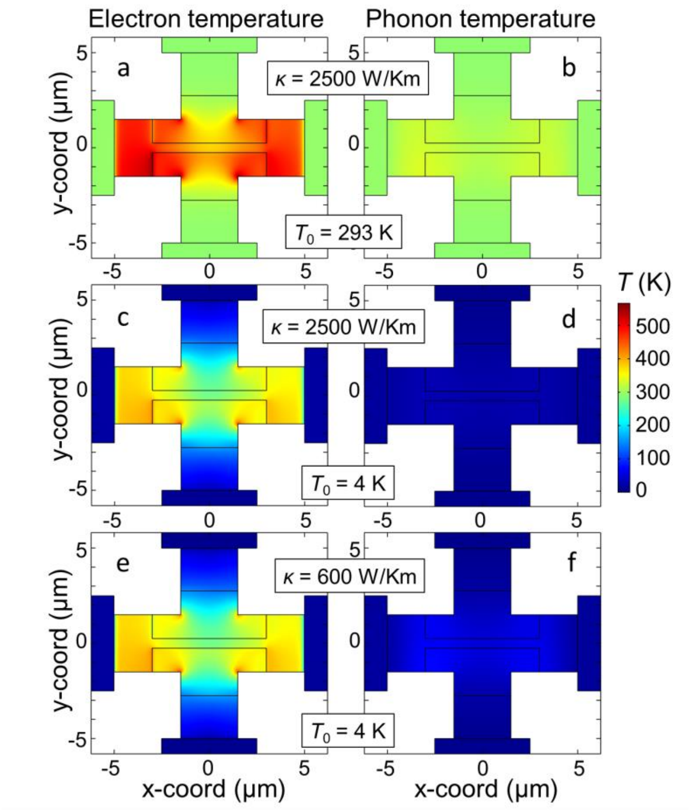

Figure 6S. False-color representation of the electron- (a, c, e) and phonon (b, d, f) temperature distributions, calculated for 1 mW of power at the ambient temperature T_0 = 293 K (a, b) and 4 K (c, d, e, f) with the phonon thermal conductivity of graphene κ = 2500 W(m K)$^{-1}$ (a, b, c, d) and κ = 600 W(m K)$^{-1}$ (e, f). The current bias is applied to the horizontal- while the TEP-voltage occurs at the vertical electrodes. No significant difference between the distributions is seen except for the phonon temperature at T_0 = 4 K (d, f).

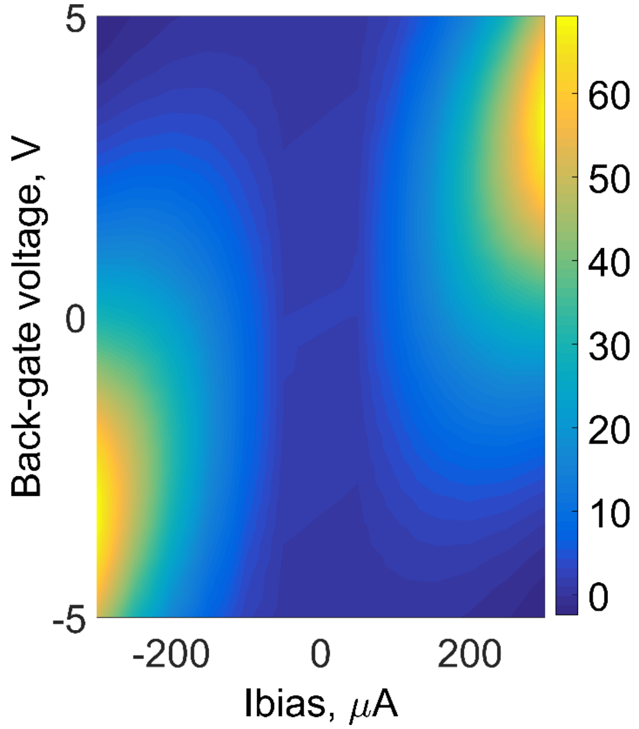

Figure 7S. COMSOL simulations of $V_{\perp}(V_{bg},I)$ including the self-gating effect (see above). The color-bar scale is in mV. The parameters used in the simulations are as follows: n_0 = 3e11 cm^{-2}; μ = 5000 cm^2(V s)$^{-1}$; V_{g1} = - V_{g2} = 3 V; C_{tg}/e = 8.14e10 V^{-1}cm^{-2}; C_{bg}/e = 7.08e10 V^{-1}cm^{-2}. $S(T,V_g)$ is calculated from the Mott formula scaled to the maximum value of 100 μV/K at T = 300 K.

4. Microwave measurements

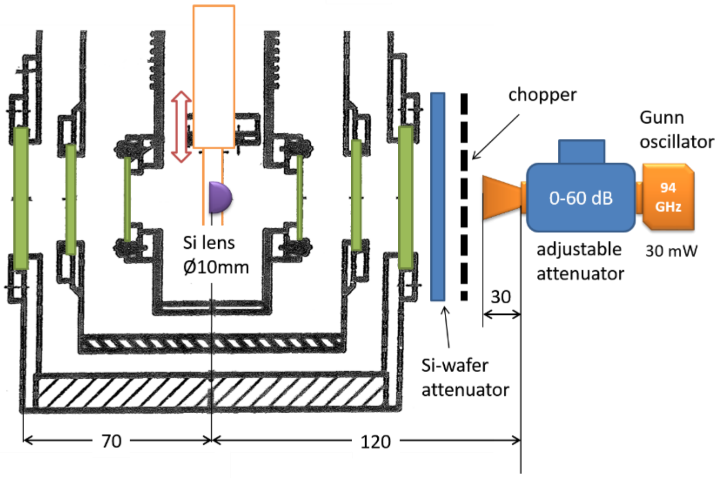

Figure 8S. Microwave measurements setup for temperatures 4 – 300 K and variable microwave power.

For the microwave measurements, we glue the sample back-to-back to a Si lens and place it into the pulse-tube cryostat (Janis PT450) having optical windows transparent for microwaves and allowing for

measurements at temperatures 4 – 300 K. The Gunn oscillator (94 GHz & 30 mW; QuinStar Technology) is connected to a horn antenna through an adjustable attenuator (0 – 60 dB). There is another attenuator made from a couple of Si wafers glued to each other at 45 degrees of relative in-plane rotation, providing a constant 20 dB attenuation, almost independent of the polarization of incident microwaves. We use a mechanical chopper at frequency 7 – 900 Hz to modulate the microwave power and synchronously detect the signal with the help of DSP SR850 lock-in amplifier from Stanford Research. Instrumentation amplifiers (APM01, 1000x gain) isolate samples from the electronics connected to a computer, without any extra filters. The absence of filtering makes the setup to be susceptible to electrical noise from e.g. compressor of the pulse-tube cryostat and other sources of noise. Nonetheless, the overall minimum noise level is still about 10 – 20 nV Hz$^{-0.5}$, only a factor two higher than the input noise of the instrumentation amplifiers at the modulation frequency.

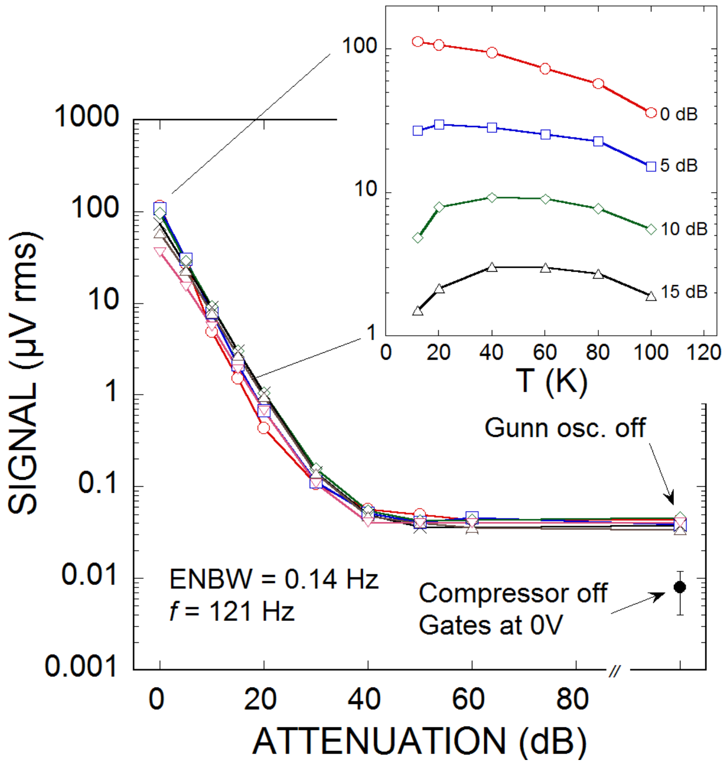

Figure 9S. The signal as a function of attenuation level of the adjustable attenuator at T = 12 – 100 K and V_{g1} = - V_{g2} = 2 V. The inset shows the temperature dependence of the signal at the four attenuation levels indicated. The chopper frequency, the lock-in time constant, and the lock-in input filter roll-off slope was 121 Hz, 0.3 s, and 24 dB/Oct, respectively. The last two parameters determine the equivalent noise bandwidth ENBW of 0.14 Hz. The singular point with error bars indicates the overall noise level of the measurement system when the compressor of the pulse-tube cryostat is switched off and the gates are short-circuited to the measurement ground.

The main results of the microwave measurements are presented in Fig. 4 of the paper. Here, we show additional measurements performed on another sample with the same layout (see Fig. 9S). In this figure, we depict the signal as a function of the adjustable attenuation level at different temperatures T = 12 – 100 K and V_{g1} = - V_{g2} = 2 V while keeping other parameters of the system constant. Initially, the dependence is linear at small attenuation. At high attenuation corresponding to the small microwave

power reaching the sample, the signal successively disappears in the electrical noise of our system. The minimum noise level can be reached when the compressor is switched off and the gates are disconnected from their voltage sources. We believe that the noise can be reduced about ten times by using better pre-amplifiers and cryogenic liquids instead of the “dry” cryostats involving powerful compressors. Furthermore, a chemical doping can replace the electrical gating thereby avoiding the noise from the voltage sources.
